# ‘It’s just a mucocele’: a case report of a massive appendiceal mucocele presenting as a left upper quadrant mass

**DOI:** 10.1308/rcsann.2022.0068

**Published:** 2023-01-09

**Authors:** CC Kearsey, S Dritsas, M Mathur, J Wild

**Affiliations:** ^1^ The Christie NHS Foundation Trust, UK; ^2^ Institute of Translational Medicine, University of Liverpool, UK

## Abstract

Tumours of the appendix are very rare tumours that can and often present with a mucocele. This is a case report highlighting the associated pathology of appendix tumours and the management of a large mucocele. Specifically, how a right hemicolectomy is very rarely needed in these cases regardless of size and local anatomical relationships and some important considerations for the practicing surgeon in the non-tertiary centre that encounters a case like this.

## Background

Tumours of the appendix are rare disorders that often present clinically as the mucocele. Surgeons will be familiar with the incidental finding on computed tomography (CT) scan of the appendix mucocele, but what is less known is the pathology that underpins appendix tumours and how to correctly manage these ensure the best outcome.^1^ This is a case report of a 58-year-old male diagnosed with a massive appendix mucocele who underwent two-stage cytoreductive surgery and heated intraperitoneal chemotherapy (HIPEC).

## Case history

**Figure 1:**
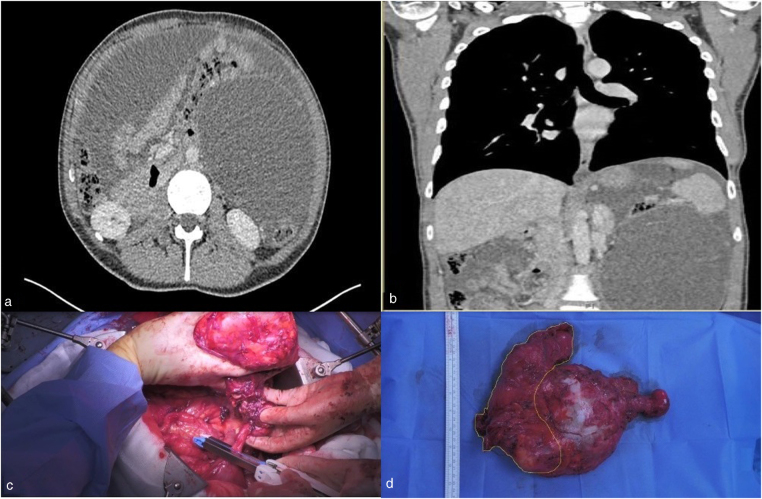
(a,b) Computed tomography scans showing a fluid-filled left upper quadrant that displaces the stomach, whole of the small bowel, spleen and kidney (a). Multiseptated collection, representing a massive appendiceal mucocele (b) coronal section of the same CT scan highlighting displacement of the stomach, spleen small bowel and kidney. (c) Intraoperative image highlighting a normal appendix base despite massive size and pseudomyxoma peritonei indicating there is rarely any need for right hemicolectomy at the time of index surgery. (d) Resected specimen showing size and a bird’s-eye view highlighting the en-bloc colon (yellow) with the rest being mucocele.

We present a 58-year-old male with no medical history presenting with upper abdominal discomfort for 1 year. No other symptoms were reported. The patient underwent CT scanning of the abdomen and pelvis (Figure 1). The CT scan identified a massive mucocele of the appendix with mucin around the spleen. The multidisciplinary team (MDT) recommended cytoreductive surgery with or without HIPEC. Mucin was drained from the abdomen in all quadrants and the mass was found to be adherent to the spleen. The base of the appendix was normal, and the densely adherent splenic flexure was resected en-bloc (Figure 1). The patient’s peritoneal cancer index score was 30/39.

A second operation to achieve complete cytoreduction was carried out following specialist MDT discussion highlighting the possible need for a two-stage procedure and confirmed owing to patient haemodynamic instability in the first procedure. Procedures included mass excision, subtotal colectomy, umbilectomy, anterolateral peritonectomies, splenectomy, cholecystectomy, greater and lesser omentectomy and ileorectal anastomosis. Histopathology showed a low-grade appendiceal neoplasm (LAMN, pT4a) arising from the appendix with acellular mucin.

## Discussion

The appendix is identical in cross-sectional histology and colonic histology; however, tumours of the appendix often have fundamental differences in terms of their molecular biology and histopathology. A combination of Peritoneal Surface Oncology Group International (PSOGI) and World Health Organization (WHO)^2^ terminology categorises tumours of the appendix as benign polyps (hyperplastic and serrated), benign tumours, such as appendiceal mucinous neoplasms, and pseudomyxoma peritonei (PMP) and variations in malignancy that include adenocarcinoma, goblet cell tumours and neuroendocrine tumours. This case report focuses solely on PMP, the pathology of the condition and further management.

These benign lesions cause a mucocele due to deposits of mucin accumulating in the obstructed appendix. They are diagnosed histologically and show a varying type of epithelial architecture, but all exhibit a lack of infiltration consistent with their benign nature. These mucinous neoplasms are categorised into two forms based on the level of atypia in the specimen. Those showing mild cytological atypia and are classified as LAMN and those with high-grade atypia and increased mitotic figures as high-grade appendiceal neoplasms (HAMN). HAMN lesions are considered to have higher rates of malignant conversion. Around 94% have KRAS mutations and in around 40% of cases, p53 protein was overexpressed, which is associated with transformation to PMP and poor prognosis. LAMNs tend to be Microsatellite instability (MSI) stable but more studies are required to fully characterise the genetics.^3^

PMP is a clinical descriptor and classed as ‘borderline malignant’ although is histologically benign. This condition is characterised by an accumulation of peritoneal mucus due to disseminated peritoneal mucinous neoplasia. The key step in progression from LAMN to PMP is the extravasation of mucin from the appendix into the peritoneum and this can exhibit a cellular component spreading along the peritoneum, or be acellular with mucinous ascites (LAMN 1 (pTis, pT3+) – no perforation; LAMN 2 (pT4a+) – perforated mucin into the peritoneal cavity). If no perforation occurs and the LAMN is pTis-pT3 then patients typically undergo a surveillance pathway consisting of CT scans at 6, 18 and 60 months, resulting in discharge if all normal. Patients with pT3 LAMN tumours are quoted a 2% lifetime risk of PMP development.^4^ Clinically, true PMP is a slowly progressing disease and PMP rarely spreads beyond the peritoneal cavity. The ‘malignant’ terminology comes from the mortality associated with intestinal obstruction due to peritoneal deposits on the small bowel, often the cause of death for most PMP patients. Review of the histological features of PMP has led to the various grading systems given in Table 1.

**Table 1:** Comparison of the pathological classifications systems of peritoneal disease/PMP and the usual primary tumour causing each one

PSOGI CONSENSUS 2015	WHO 2020 (TNM8)	Usual primary tumour
Acellular mucin	Not classified [M1a]	LAMN
Low-grade mucinous carcinoma peritonei	MCP Grade 1 [M1b]	LAMN
Intermediate-grade mucinous carcinoma peritonei	Not classified [M1b]	LAMN
High-grade mucinous carcinoma peritonei	MCP Grade 2 [M1b]	HAMN
		Non-mucinous AC
		Mucinous AC
		Goblet cell AC
High-grade mucinous carcinoma peritonei with SRCs	MCP Grade 3 [M1b]	SRC AC
		Goblet cell AC

AC = appendix carcinoma; HAMN = high-grade appendix mucinous neoplasia; LAMN = low-grade appendix mucinous neoplasia; M1a = acellular mucin; M1b = intraperitoneal metastasis; M1c = extraperitoneal metastasis; MCP = mucinous carcinomatosis peritonei; PSOGI, Peritoneal Surface Oncology Group International; SRC = signet ring cells; WHO = World Health Organization.

### Cytoreductive surgery and HIPEC

Cytoreductive surgery has been widely accepted as an effective method of treating peritoneal disease and is usually combined with the application of HIPEC. This involves removal of all macroscopic deposits in the form of visceral resection and peritoneal stripping. Often these are bowel resections (large and small), cholecystectomy, splenectomy, liver capsulectomy, omentectomy, bilateral salpingo-oophorectomy and peritonectomy in sites such as the hemidiaphragm. HIPEC is ineffective against nodules that are larger than 2.5mm, therefore all nodules ≥2.5mm must be resected for the treatment to be efficacious. The folds and reflections of the peritoneal cavity can harbour fluid effectively, which creates the need for opening/removing the lesser omentum and falciform ligament to ensure adequate flow of chemotherapy around the peritoneal cavity.^5^ The procedure is carried out by trained technicians who calculate the volumes required and set up the specialist equipment used in the administration, supervised by the overseeing surgeon. The dispensing equipment calculates the flow rate and four temperature probes placed intra-abdominally ensure that the chemotherapy is kept as close to 42°C as possible.^5^ The current regime of choice in the case of appendix tumours is mitomycin C.

## Conclusions

### Key messages for managing the ‘unusual’ appendix

When managing the unusual appendix in a local hospital, some key points will ensure the best outcome for these rare tumours.

1. Early specialist centre referral is required if possible; appendix tumours are rare and should be managed long term in specialist centres that have a high case volumes.
2. If a mucocele or suspected appendix tumour is found incidentally in the emergency department, then perform an appendectomy as normal. Avoid transecting obvious tumours and resect on the caecal pole if needed.
3. If intra-abdominal mucin is present, either in the right iliac fossa or throughout the abdomen, sample this for histology. This will identify the appendix tumour and give an indication of the prognosis for the patient.
4. Once the patient has recovered, they can be followed up by the specialist centre of choice because, if needed, completion right hemicolectomy is best performed in conjunction with cytoreductive surgery and HIPEC.
